# Household practices related to disease transmission between animals and humans in rural Cambodia

**DOI:** 10.1186/s12889-015-1811-5

**Published:** 2015-05-09

**Authors:** Kristina Osbjer, Sofia Boqvist, Seng Sokerya, Chheng Kannarath, Sorn San, Holl Davun, Ulf Magnusson

**Affiliations:** Division of Reproduction, Department of Clinical Sciences, Swedish University of Agricultural Sciences (SLU), Box 7054, SE-750 07 Uppsala, Sweden; Department of Biomedical Sciences and Veterinary Public Health, SLU, Uppsala, Sweden; Centre for Livestock and Agriculture Development, Phnom Penh, Cambodia; National Institute for Public Health, Phnom Penh, Cambodia; National Veterinary Research Institute, Phnom Penh, Cambodia

**Keywords:** Zoonoses, Cambodia, Livestock, Rural household, Household practice

## Abstract

**Background:**

Zoonotic diseases are disproportionately affecting poor societies in low-income countries and pose a growing threat to public health and global food security. Rural Cambodian households may face an increased likelihood of exposure to zoonotic diseases as people there live in close association with livestock. The objectives of the study was to identify practices known to influence zoonosis transmission in rural Cambodian households and relate the practices to agro-ecological region, socio-economic position, demographics, livestock management and zoonosis awareness.

**Methods:**

The study was conducted in three different agro-ecological regions of Cambodia; 10 villages each in the central lowlands, north-west wetlands and on the south coast, where information was obtained in questionnaires administered to 300 households, and 30 village heads and animal health workers.

**Results:**

Descriptive analysis revealed a gender difference in responsibility for livestock and that the main purpose of raising livestock was for sale. Few respondents (6%) perceived a likelihood of disease transmission in their village between livestock, humans and wildlife, despite household practices related to zoonosis transmission being common. More than one-forth of households practised behaviours such as culling sick animals for consumption, eating animals found dead and allowing animals to enter sleeping and food preparation areas. Associations between household practices and possible explanatory factors were analysed with multivariable models using generalised estimation equations to account for clustering of practices within villages. Factors found to influence household practices were agro-ecological region, socio-economic position, number of people in the household, livestock species reared and awareness of zoonoses.

**Conclusions:**

Cambodia has experienced numerous fatal human cases of zoonotic influenza and extensive influenza information campaigns have been run, yet only a few of the households surveyed here reported the threat of zoonosis to be a concern in their village. Zoonosis awareness was positively related to hand washing behaviour, but other practices associated with an increased or decreased likelihood of exposure to zoonotic pathogens were unaffected by awareness. The findings indicate a knowledge-to-action gap among rural farmers and highlight the necessity for reconstructed interventions in zoonotic disease control.

**Electronic supplementary material:**

The online version of this article (doi:10.1186/s12889-015-1811-5) contains supplementary material, which is available to authorized users.

## Background

Zoonotic diseases, naturally transmissible between animals and humans, make up more than 60% of emerging infectious diseases (EIDs) in humans [1] and are regarded as posing a growing threat to public health and global food security [2]. Zoonotic diseases are estimated to cause about a billion cases of illness in people and millions of deaths every year and disproportionally affect low-income countries, with the poorest within society affected the most [3]. The true public health and economic impact of zoonotic diseases are most likely underestimated, mainly due to under-reporting of disease events [4].

Southeast Asia has been identified as a hotspot for EIDs, in particular zoonotic diseases, as a result of many factors, including population growth, urbanisation, political and social disruption, agriculture and livestock intensification, deforestation, and climate change [5]. The region has seen the emergence of several recent epidemics, such as Severe Acute Respiratory Syndrome (SARS), Highly Pathogenic Avian Influenza H5N1 and pandemic influenza A (H1N1/2009) [5,6]. Cambodia, which is among the poorest countries in Southeast Asia, has a population of 15 million, with 80% living in rural areas [7]. Resource-scarce smallholder farmers represent the majority of agricultural producers [8] and livestock are traditionally raised in a mixed farming system [9]. Close interaction between livestock and humans is enabled by free ranging poultry and livestock pens bordering the house, allowing animals to access cooking and sleeping areas. In these households access to health and veterinary services is limited and household practices associated with an increased likelihood of exposure to zoonotic pathogens are frequent [9]. One study in Cambodia showed that inadequate hand washing and slaughtering of poultry were risk factors for H5N1 virus infection in humans [10]. Other studies in several countries have found that consumption of undercooked meat is a major risk factor for human infection with *Toxoplasma gondii* [11], while a study in Canada identified associations between zoonotic disease transmission and feeding animals raw meat [12]. Several factors affecting household practices have been identified. These include risk perception, agro-ecological conditions [13], household demographics [14], cultural aspects [15], level of education and socio-economic position of the household [16]. There is, however, a need for a thorough understanding about these factors and how they are interrelated. Such knowledge can guide extension services in achieving more effective zoonosis control.

The objective of the study, which was carried out in Cambodia, was to identify practices known to influence zoonosis transmission in rural households and relate these practices to the agro-ecological region, socio-economic position, demographics, livestock management and zoonosis awareness.

## Methods

### Study sites

**Figure 1:**
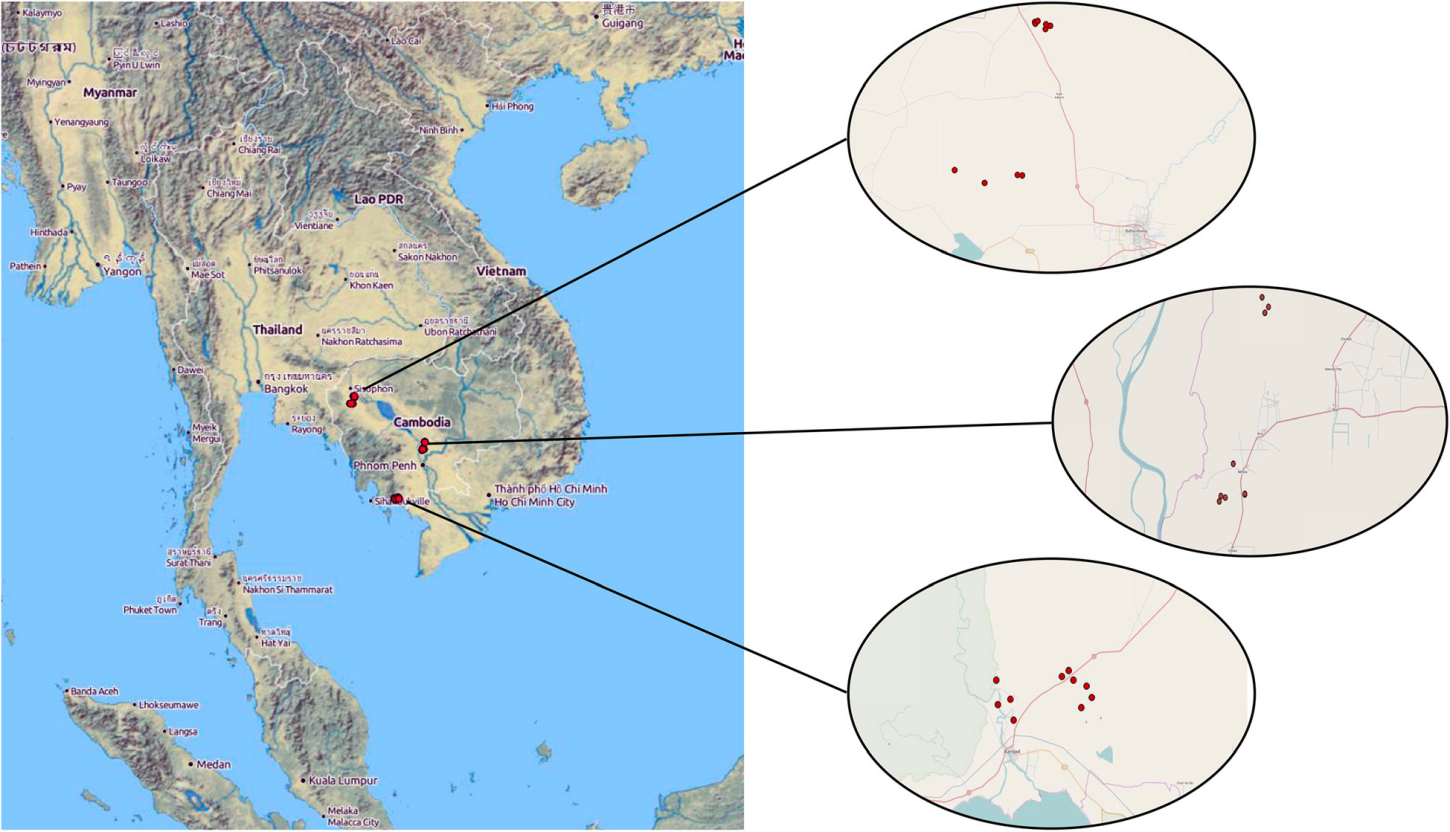
Geographical distribution of the 30 villages included in the cross-sectional study (Cambodia 2011–2013). © OpenStreetMap contributors (openstreetmap.org).

The study involved three out of Cambodia’s four agro-ecological regions, to cover possible differences in climate, farming traditions and culture. These regions were: Kampong Cham province, a lowland area characterised by fertile cultivated plains close to the Mekong river; Battambang province, characterised by immense wetlands resulting from flooding which have substantial biological diversity and border Lake Tonle Sap [17]; and Kampot province, a coastal area dependent on fish and containing the wildlife-rich Preah Monivong Bokor National Park (Figure 1).

### Study design and data collection

Data were collected on 10 days per region, with Kampong Cham province visited in May 2011, Battambang province in July 2012 and Kampot province in March 2013. In each region, 10 villages were included and each village was visited for one full day. The number of villages was decided based on practical and economic considerations for sample collections as this study formed part of a larger research project on zoonotic diseases. Selected villages were those best meeting the following three criteria: the village had to be situated within 5 km from a main road; it had to have various species of livestock; and there had to be interactions between humans, domestic animals and wildlife. Within each village, the 10 households keeping as many different livestock species as possible according to the village animal health worker and village head were selected as a purposive sample. A total of 30 villages and 300 households in the three regions were included. The target number of households was calculated based on requirements for the larger research project on zoonotic diseases and was based on sample size for expected disease prevalence, with addition of 15% to adjust for possible confounding and interaction in the statistical modelling [18]. The number of households included also ensured that the minimum requirements for “qualitative health research” were met [19]. Geographical position at the central point of the villages included in the study was recorded using a handheld global positioning system (GPS; Garmin eTrex H).

Two questionnaires were developed: i) a village questionnaire targeted at the village head and animal health worker, with questions on development support and livestock management; and ii) a household questionnaire, targeted at the female head of the household, with questions on household practices related to zoonosis transmission (Table 1), as well as socio-economic position, demographics, livestock management and zoonosis awareness. The household questionnaire targeted the female head, as women are traditionally, and to a larger extent than men, responsible for day-to-day household duties and subsistence farming in Cambodia [20]. The questionnaires, which each took around 40 minutes to complete, consisted of open, closed and semi-closed questions (two-choice and ranking questions), with some probing questions to clarify the answers. The questionnaires were pre-tested in two villages in a non-participating province and adjusted according to input before the study began. The household questionnaire also included validation questions, which allowed questionnaires to be checked for internal consistency. The households and villages included were allocated a code. Interviews were conducted in Khmer and all data were checked for accuracy by the team leader. Prior to the interviews, village heads and participating household members were informed about the study per se, that participation was voluntary and that their identity should not be disclosed. People selected for an interview were asked for verbal consent before the interviews were conducted, and were given a project T-shirt and bar of soap at the end, as a thank you for their involvement.

**Table 1:** **Self-reported household practices by province in three different agro-ecological regions (n = 300)**

**What do you practise in this household?**	**KPC**	**BB**	**KT**	**All regions**
	**%**	**%**	**%**	**% (n)**
Eat undercooked meat	7	12	4	8 (23)
Feed your livestock uncooked meat waste	3	25	27	18 (55)
Cull sick animals for consumption	24	29	30	28 (83)
Eat animals found dead	40	18	27	28 (85)
Wash hands with soap before and after cooking	85	85	98	89 (268)
Wash hands with soap after handling live animals	71	82	98	84 (251)
Keep live animals away from sleeping and food preparation areas	43	74	98	72 (215)
Bury or burn meat waste products	79	80	83	81 (242)
Daily collection of manure indoors and outdoors	80	90	90	87 (260)
Capture and slaughter wild animals for consumption	11	11	2	8 (24)
Slaughter domestic animals	76	71	44	64 (191)

Kampong Cham province (KPC), n = 100; Battambang province (BB), n = 100; and Kampot province (KT), n = 100, (Cambodia 2011–2013).

Each village was visited by a survey team that entailed 10–12 members and was led by the author KO. The team consisted of staff from the National Veterinary Research Institute in Phnom Penh; final year students from the Preak Leap National School of Agriculture, Phnom Penh, and the Royal University of Agriculture, Phnom Penh; and district and commune livestock officers in the study regions. The team was trained for one full day prior to the field work to ensure that the questionnaires and the aims of the study were fully understood.

The questionnaires are provided as additional files. The village questionnaire (Additional file 1): Zoonoses in humans and livestock in rural Cambodia – Village questionnaire) and the household questionnaire (Additional file 2: Zoonoses in humans and livestock in rural Cambodia – Household questionnaire).

#### Development support

Each village that participated in the study had ongoing externally supported development projects. In Kampong Cham province, 22 different development projects supporting livestock management and human health improvements were reported as ongoing. Battambang and Kampot provinces each had five different development projects ongoing supporting livestock management and human health improvements. The projects were run by the Cambodian Government, international organisations and non-government organisations. The reported ongoing projects were of similar size and type in the three regions.

### Assessment of household socio-economic position

A wealth index based on household land ownership, household dwelling and household ownership of consumer durables was calculated to define the socio-economic position of participating households [21]. This was done by collecting information on eight self-reported household belongings (Table 2). Households with the listed belongings were given a score of one for each of the eight belongings. To get a final indicator each belonging was then multiplied by a weighting factor of 1–2. The weighting factor was based on previous research in the region where housing construction and access to safe water was identified as more closely linked to the socio-economic position of the household than ownership of livestock and consumer durables [22,23]. The final wealth index was calculated as the sum of all indicators with a maximum score of 11.

**Table 2:** **Self-reported household belongings and weighting factors used in calculation of the household wealth index, (Cambodia 2011–2013)**

**Household belonging**	**Weighting factor**
All farming land owned by the household	1
House construction - concrete or brick	2
Roof construction - tiled	2
Safe water as main water source^1^	2
TV in the household	1
Cell phone in the household	1
Vehicle or machine owned by the household^2^	1
Cattle or water buffalo owned by the household	1

^1^Safe sources are bottled water, and boiled or filtered water from: well, pond, stream or rainwater.

^2^Bicycle, motorcycle, car, hand tractor, ox chart, rice miller or pumping machine.

### Data management and statistical analysis

Data collected were independently translated by two translators from Khmer into English and compared for consistency before being transcribed into spreadsheets in Microsoft Office Excel 2010. Statistical analysis was performed in SAS for Windows 9.3 (SAS Institute Inc., Cary, NC). Descriptive statistics were calculated to define demographic characteristics and livestock management. A one-way analysis of variance (ANOVA) was used to test the difference in means of the wealth index between regions. Spearman’s rank correlation coefficient was used as an exploratory tool to test putative relationships between household practices and agro-ecological region, socio-economic position, number of people in the household, number and species of livestock reared in the household, and awareness of zoonoses. All variables were further analysed by a multivariable logistic regression analysis using generalised estimation equations to account for clustering of practices within villages.

Models were built to investigate associations between household practices related to zoonosis transmission and possible explanatory factors selected on the basis of prior knowledge of possible confounders and potential influence on household practices. One model was built for each of the household practices, with the practices as interchanging response variables against all the explanatory household factors: agro-ecological region; socio-economic position; number of people in the household; whether there were children in the household; number of chickens, ducks, other avian species, pigs, cattle and buffalo; whether the respondent knew of any zoonotic diseases; and whether the respondent perceived a likelihood of zoonoses in the village. Village was added to all models for the working correlation of the generalised estimating equations analysis to account for clustering of repeated measures within village, as nested within region.

All models were applied using backward removal of variables with a p-value of ≤0.2, with this higher p-value chosen to avoid early exclusion of variables that might influence the model [24]. Manual backward step-down selection was then applied at a p-value of ≤0.05. Confounding was controlled for by including omitted variables that changed the estimate of the other variables by more than 20%. Two-way interactions between all explanatory factors were investigated. The statistical significance level was defined as a two-tailed p-value ≤0.05.

QGIS 2.0.1 software was used to map the distribution of villages in © OpenStreetMap contributors (openstreetmap.org).

### Study Approval

Ethical approval (43 NECHR, 8^th^ April 2011) was obtained prior to the survey from the National Ethics Committee for Health Research, Ministry of Health in Cambodia, and an advisory ethical statement (Dnr 2011/63) was obtained from the Regional Board for Research Ethics in Uppsala, Sweden.

## Results

### Descriptive results

#### Demographic characteristics and socio-economic position of households

**Figure 2:**
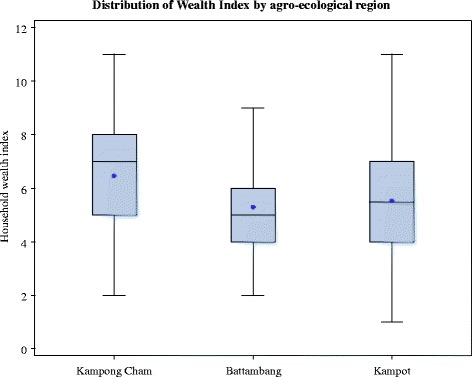
Boxplot showing household wealth index (calculated based on eight self-reported household belongings) by the three different agro-ecological regions (n = 300): Kampong Cham province (n = 100); Battambang province (n = 100); and Kampot province (n = 100), (Cambodia 2011–2013).

The median household size in the households investigated was 5.0 (range 1–17), with a mean of 5.7 (Standard Deviation (SD) 2.1), where a household was defined as a group of people making common arrangements for food and shelter. The mean wealth index for the three regions was 6.4 (SD 2.0) in Kampong Cham, 5.3 (SD 1.5) in Battambang and 5.5 (SD 2.2) in Kampot. The difference in mean wealth index between the regions was significant (p = 0.0001), with Kampot province showing the widest range of index scores (Figure 2).

#### Livestock management

The village-level questionnaires indicated that 84% of all households in the villages surveyed raised poultry (chicken and ducks), while 28% had pigs and 38% ruminants (cattle and buffalo). Of the 300 households that were actually visited in the study, 296 (99%) raised livestock. The four households without livestock were situated in Kampong Cham province and had recently lost or sold their livestock. Participating households in Kampot province raised more livestock than households in the other two provinces: 100% raised chickens, 82% pigs and 78% cattle (Table 3). In Kampong Cham province, buffalo were more common, being present in 23% of households compared with 9% of the households in Kampot province and in none of the households in Battambang province. Poultry were in general raised in larger numbers than cattle, pigs and buffalo.

**Table 3:** **Frequency of number of animal species in the households surveyed in three different agro-ecological regions (n = 300)**

		**KPC**	**BB**	**KT**	**All regions**
	**Frequency**	**%**	**%**	**%**	**% (n)**
No. of chickens	0	20	5	0	8 (25)
	1-10	36	38	45	40 (119)
	11-20	19	27	35	27 (81)
	≥21	25	30	20	25 (75)
No. of ducks	0	74	59	60	64 (193)
	1-10	18	25	35	26 (78)
	11-20	4	9	4	6 (17)
	≥21	4	7	1	4 (12)
No. of pigs	0	60	59	18	46 (137)
	1-2	18	10	43	24 (71)
	3-5	10	10	25	15 (45)
	≥6	12	21	14	16 (47)
No. of cattle	0	43	40	22	35 (105)
	1	52	52	72	59 (176)
	2-3	5	5	5	5 (15)
	≥4	0	3	1	1 (4)
No. of buffalo	0	77	100	91	89 (268)
	1-2	5	0	7	4 (12)
	3-5	15	0	2	6 (17)
	≥6	3	0	0	1 (3)

Kampong Cham province (KPC), n = 100; Battambang province (BB), n = 100; and Kampot province (KT), n = 100), (Cambodia 2011–2013).

The livestock housing system differed between livestock species. Pigs were raised in a free range system by 20% (63/163) of the households who kept pigs, while 99% (272/275) and 71% (153/215) of the households with poultry and ruminants, respectively, raised them entirely free ranging or free ranging and confined combined. The responsibility for poultry, pigs and ruminants was shared between women, men and children in about 40% of the households (Table 4). In the remaining households, women took more responsibility for poultry and pigs and men for ruminants. The most common reasons for raising poultry were reported as sale and family consumption (Table 4). The main purpose of raising pigs and ruminants was sale, to earn an income.

**Table 4:** **Responsibility for livestock and purpose of livestock production in the studied households, (Cambodia 2011–2013)**

	**Poultry**	**Pigs**	**Ruminants**
**Variable**	**n (%)**	**n (%)**	**n (%)**
**Livestock responsibility**	n = 283	n = 165	n = 220
Women	115 (41)	86 (52)	31 (14)
Men	51 (18)	19 (12)	83 (38)
Children	5 (2)	-	10 (5)
Mixed	112 (40)	61 (37)	96 (43)
Purpose of livestock production*	n = 282	n = 163	n = 218
Sale	234 (83)	154 (94)	174 (80)
Emergency sale	26 (9)	26 (16)	47 (22)
Family consumption	218 (77)	5 (3)	-
Cock fighting	2 (1)	-	-
Draught power	-	-	45 (21)
Dowry and heritage	-	-	3 (1)

*Multiple purposes reported by each household.

#### Likelihood and knowledge of zoonoses

Only 6% of households regarded disease transmission between livestock, humans and wildlife as likely within their village, although 69% knew of a disease transmissible between animals and humans. Avian influenza was mentioned as a zoonosis by 65% of all households and swine influenza, diarrhoea, tuberculosis or rabies were also mentioned, each by less than 5% of households.

### Analyses of self-reported household practices

#### Associations between household practices and explanatory factors

Each of the household practices presented in Table 1: Self-reported household practices by province in three different agro-ecological regions, were analysed in a separate model for associations with potential explanatory household factors and confounders. All response variables except eating undercooked meat, and capturing and slaughtering wild animals for consumption were associated with at least one explanatory factor. Models with significant associations between household practices and explanatory factors are presented in Table 5. Feeding animals uncooked slaughter waste was associated with region. This practice was also more frequently reported in households where the respondent knew of a zoonosis and where they perceived a likelihood of zoonosis transmission between wildlife, livestock and humans in the village. Eating animals found dead was associated with region. The factor number of buffaloes was not significantly associated with the practice to eat animals found dead, but was correlated to province (p = 0.007) and changed the model estimate by more than 20% when removed from the model, suggesting that number of buffaloes was a confounder in the model. Washing hands before and after cooking was associated with region and was more commonly reported in households where the respondent knew of any zoonosis. The related practice of washing hands with soap after handling live animals was also associated with region and was similarly increased in households where the respondent knew of any zoonosis. Keeping animals away from sleeping and food preparation areas was associated with region and was more common in households with a lower wealth index. Burning or burying meat waste products was associated with a higher number of people in the household. Daily collection of manure indoors and outdoors was associated with households rearing more cattle. Finally, the practice of slaughtering domestic animals was associated with region and was found to be more common in households with a higher number of people, rearing more chickens and in households where the respondent knew of any zoonosis.

**Table 5:** **Association between the response variable household practice and the explanatory factors: agro-ecological region, socio-economic position** ^**1**^ **, number of people in the household** ^**1**^ **, number and species of livestock reared** ^**1**^ **, and zoonosis awareness (n = 300)**

**Household practice** ^**2**^	**Explanatory factors** ^**2**^	**OR (95% CI)**	**P-value**
Feeding animals uncooked slaughter waste	Region	-	0.0009
	KPC vs KT	9.6 (3.5-26)	<0.0001
	KPC vs BB	12 (4.5-32)	<0.0001
	Have knowledge of zoonoses	2.2 (1.0-4.5)	0.04
	Perceive likelihood of zoonoses	7.5 (2.2-26)	0.001
Eating animals found dead	Region	-	0.04
	KPC vs KT	ns	ns
	KPC vs BB	0.4 (0.2-0.7)	0.003
	No. of buffalo^3^	1.3 (0.9-1.7)	0.14
Washing hands with soap before and after cooking	Region	-	0.02
	KPC vs KT	ns	ns
	KPC vs BB	ns	ns
	Have knowledge of zoonoses	1.4 (1.3-7.3)	0.01
Washing hands with soap after handling live animals	Region	-	0.02
	KPC vs KT	ns	ns
	KPC vs BB	ns	ns
	Have knowledge of zoonoses	1.4 (1.3-7.3)	0.01
Keeping live animals away from sleeping and food preparation areas	Region	-	0.0002
	KPC vs KT	67 (9.5-470)	<0.0001
	KPC vs BB	2.9 (1.5-5.5)	0.0013
	Wealth index	0.8 (0.7-0.9)	0.0003
Burying or burning meat waste products	No. of people in household	1.3 (1.1-1.5)	0.01
Daily collection of manure indoors and outdoors	No. of cattle	1.2 (1.0-1.5)	0.01
Slaughtering domestic animals	Region	-	0.0007
	KPC vs KT	0.2 (0.1-0.3)	<0.0001
	KPC vs BB	ns	ns
	No. of people in household	1.5 (1.1-1.3)	0.002
	No. of chicken	1.0 (1.0-1.1)	0.004
	Have knowledge of zoonoses	1.9 (1.1-3.2)	0.02

^1^Quantitative explanatory factor.

^2^Only significant (p < 0.05) response variables and explanatory factors from the logistic analysis shown.

^3^Buffalo retained in the model despite a non-significant p-value, as removal caused a change in the province estimate of more than 20%.

Kampong Cham province (KPC), Kampot province (KT), Battambang province (BB), (Cambodia 2011–2013).

No significant interactions or correlations, apart from the one presented, were found between the explanatory factors.

## Discussion

Understanding the factors governing transmission of zoonoses in rural Southeast Asian settings is important given the regional zoonosis emergence. This study showed that despite knowledge of zoonoses, few respondents in the rural Cambodian households surveyed perceived a likelihood of disease transmission between livestock, humans and wildlife in their village and many households carried out practices associated with an increased likelihood of exposure to zoonotic pathogens. The study also identified associations between household practices linked to zoonosis transmission and the household’s agro-ecological region, socio-economic position, number of people in the household, species and numbers of livestock reared and zoonosis awareness. Lastly was a clear gender division in responsibility for livestock found and a divergence was observed in the purpose behind rearing different livestock species.

Household practices analysed in this study were selected from previously described practices related to zoonosis transmission with the aim of covering various transmission routes for pathogens known to pass between humans, livestock and wildlife in low-biosecurity backyard farming systems [10,12,25-27]. Information on occurrence of household practices was obtained through self-reporting, which is less intrusive than structured observation and can be carried out with a single household visit. While being well aware of the possibilities for under-reporting of hazardous behaviour and over-reporting of good hygiene practices due to intentionally or unintentionally perceived desirable responses, we opted for the self-reporting methodology to enable inclusion of a larger number of households [28,29]. Validation questions on household practices were included in the household questionnaire as a precaution to minimise bias. Replies to ordinary questions and validation queries matched well, confirming the legitimacy of responses.

We found the household practice of burning or burying meat waste products to be associated with a higher number of people in the households which possibly could be explained by the need, in crowded households, to effectively dispose of a larger volume of household waste. The socio-economic position has in other studies been shown to influence precautionary household practices as a better economic condition allows upgrading of housing, sanitation and purchase of hygiene products [16,30]. Such associations were in our study not found. Instead was the practice of chasing animals away from sleeping and food preparation areas associated with a lower wealth index. A possible explanation could be that animals easily can enter cooking and sleeping areas in poor households with an open housing construction. Households with a lower wealth index will thus actively have to chase away animals while in the wealthier households the more solid housing construction used will keep animals out. The practice of chasing animals away from sleeping and food preparation areas was also associated with the agro-ecological region of the household. The regional associations identified for most of the household practices studied here may partly be explained by the different farming challenges deriving from climatic and physical conditions in the different agro-ecological regions. The results presented here, however, are likely to move beyond agro-ecology and, among other factors, also depend upon regional differences in socio-economic opportunities and development support. We believe that one explanation to the high average wealth index in Kampong Cham might be that the villages in that province had more than twice as many development support projects ongoing. Regional differences in the households’ socio-economic position could not, however uniformly explain the differences in practices between regions. Households in Kampong Cham province had the highest average wealth index, but precautionary household practices were not reported more frequently there than in the other two regions.

In all, 65% of the respondents in this study mentioned Avian Influenza as a disease transmissible between animals and humans. Awareness of Avian Influenza can possibly be explained by the nation-wide influenza awareness activities and development support in Cambodia, resembling those reported in the study villages. Remarkably, the threat of zoonoses was not reported to be a concern for the households surveyed and only a small proportion of the respondents considered disease transmission between livestock, humans and wildlife to be likely in their village. These results should be seen in the light of 42 poultry outbreaks and 56 confirmed human cases of Highly Pathogenic Avian Influenza (H5N1) reported from Cambodia between 2003 and 2014 [31,32]. Influenza information campaigns have been regularly run in Cambodia since 2004 and several studies have reported raised awareness of human-animal disease transmission among the rural population. Despite this, practices associated with zoonosis transmission persist [33-35]. Thus messages provided on disease control apparently only partially penetrate to the level of farm practices. Previous studies have revealed that simply increasing farmers’ knowledge is insufficient to change farmers’ behaviour [36]. In this study we showed that more than 25% of the households practised behaviours such as culling sick animals for consumption, eating animals found dead and allowing animals to enter sleeping and food preparation areas. A positive effect of zoonosis knowledge was associated with the practice of washing hands before cooking and after handling live animals, yet a contrasting association was found for some other practices. Feeding animals uncooked slaughter waste and carrying out slaughter was increased in households where the respondent had knowledge of zoonoses. In line with other studies in the region, our results indicate a knowledge-to-action gap [33,35]. Some understanding of the rationale behind practices may be found in the theory of planned behaviour [37]. It suggests that attitudes towards behaviours and subjective norms are among key components determining behaviour and that both attitude and norms are influenced by various background factors. In this study, apart from zoonosis awareness and socio-economic position, such factors were identified as being: agro-ecological region of the household, household size and livestock species reared. Larger households and households with a greater number of chickens were more likely to carry out slaughter, while daily collection of manure was increased in households with cattle.

Livestock management is known to be a key contributor to food security and nutrition in rural settings, but poor control of zoonoses poses a threat to human health and to livestock productivity [38,39]. When discussing risk mitigation and preventive measures for zoonotic diseases, it is important to understand the characteristics of rural livestock production, such as purpose and gender roles. Here we found that while ruminants play an important role in producing draught power and poultry are often kept for family consumption, the predominant purpose of raising poultry, pigs and ruminants was sale. Women took the main responsibility for the homestead species (poultry and pigs) and men for ruminants, which is a common division of labour in low-income countries [40,41]. Other studies have shown that decisions regarding household practices may not be evenly distributed between women and men [33]. Thus understanding and considering gender dynamics within the household should be a primary consideration in the development of zoonosis control programmes. Interventions may also be directed towards certain target groups depending on livestock species and their contribution to livelihoods.

Households were selected for inclusion in this study based on a set of criteria rather than random sampling. Caution is needed when generalising the results to the rural Cambodian population, as the selection was based on households with many different livestock species in easy accessible parts of three agro-ecological regions. This sampling method may have resulted in a selection bias. We believe, however, that our sample can serve as an approximation of a population-based design for species-diverse households, as the study involved a considerable number of households in 30 different villages. The study targeted female heads of the households, which may have influenced some of the responses and caused a bias towards homestead livestock species, which are traditionally cared for by women. The emphasis on women may also be reflected in the level of zoonosis awareness, as illiteracy is more prevalent in women and extension activities tend to have been targeted towards men in the past [20,42]. We also considered possible confounders due to seasonal variations, as data were collected during the hot season (May and March) in Kampong Cham and Kampot province and during the rainy season (July) in Battambang province. Seasonal differences, however, are likely to have had a minor impact on the results presented.

## Conclusions

Cambodia has experienced numerous fatal human cases of zoonotic influenza (H5N1/H1N1) and extensive influenza information campaigns have been run, yet only a few of the households surveyed here reported the threat of zoonosis to be a concern in their village and household practices linked with zoonotic disease transmission were common. Agro-ecological region, socio-economic position, livestock species reared and zoonosis awareness were factors found to be associated with household practices. Zoonosis awareness was positively related to hand washing behaviour, but other practices associated with an increased or decreased likelihood of exposure to zoonotic pathogens were unaffected by awareness. The findings indicate a knowledge-to-action gap among rural farmers and highlight the necessity for reconstructed interventions in zoonotic disease control.

## Additional files

Additional file 1:
**Zoonoses in humans and livestock in rural Cambodia – Village questionnaire.**
Additional file 2:
**Zoonoses in humans and livestock in rural Cambodia – Household questionnaire.**
