# Supplementary material for: Household practices related to disease transmission between animals and humans in rural Cambodia
Source: BMC Public Health. 2015 May 9;15:476. doi: 10.1186/s12889-015-1811-5 (PMC4427931; doi:10.1186/s12889-015-1811-5)
Supplement: Additional file 1: — Zoonoses in humans and livestock in rural Cambodia – Village questionnaire. [file 12889_2015_1811_MOESM1_ESM.pdf]

Zoonoses in Humans and Livestock in Rural Cambodia
Village/Site questionnaire

(Date today):____________________________ **Name of enumerator: ____________________________**

Person to revise this questionnaire: ____

1. Village/Site Name and Location Descriptors

(Village/Site Name):	(Commune):	(District):	(Province):
(GPS coordinates/elevation):			(Village code):
(Distance to nearest markets):			(Is there a school in the village)? ☐ Yes ☐ No
(How many entry points to/from the village are there)?			

2. Village/Surroundings People and Demographics

(Total HH in village/surroundings):	(Ethnicity):	
(Male population >15 years):	(Female population >15 years):	(Children 15 years and below):
(Major occupancies of adult population):	(Average monthly income per household):	
(Main village crops):		
(Development support activities in the area – Government, international and NGOs. Include general and human/livestock health related activities):		

3. Human Health

3.1 How often are there episodes of severe disease in humans in this village?

- ☐ At least once a year
- ☐ Once every 2 –3 years
- ☐ Every 5 years
- ☐ Other, specify………………………………………………………………………….

3.2 How often are there episodes of less severe disease i.e. respiratory diseases, gastrointestinal diseases etc?

- ☐ At least once a year
- ☐ Once every 2 –3 years
- ☐ Every 5 years
- ☐ Other, specify…………………………………………………………………………..

3.3 What season, if any, do outbreaks of disease usually occur?

……….

3.4 What are the major diseases seen and confirmed in the village?

....

....

3.5 What is the main source of water used for drinking?

For humans	For animals	
☐	☐	Well, untreated
☐	☐	Well, treated. Specify how ....
☐	☐	Nearby river or stream, untreated
☐	☐	Nearby river or stream, treated. Specify how ....
☐	☐	Pond, untreated
☐	☐	Pond, treated
☐	☐	Bottled water
☐	☐	Other, specify …………………...

3.6 Are there any routine treatments in the village (such as use of vaccines)?

- ☐ No
- ☐ Yes, for what diseases and to what group of individuals?

…………………………..

4. Livestock System

(HH with pigs):	(HH with cattle/buffalo):	(HH with chickens/ducks):
(Total No. of pigs):	(Total No. of cattle):	(Total No. of buffaloes):
(Total No. of chickens):	(Total No. of ducks):	(Total No. of other avian species, specify):

4.1 How often are there episodes of severe disease in livestock in this village causing high mortality?

- ☐ At least once a year
- ☐ Once every 2 –3 years
- ☐ Every 5 years
- ☐ Other, specify…………………………………………………………………………

4.2 What season, if any, do outbreaks of disease in livestock usually occur?

……

4.4 What are the major livestock diseases seen and confirmed in the village?

....

....

4.5 Are there any routine animal health treatments in the village (such as, use of vaccines or anthelmintics)?

- ☐ No
- ☐ Yes, what & when ……………………………………………………………………………
