# Supplementary material for: Household practices related to disease transmission between animals and humans in rural Cambodia
Source: BMC Public Health. 2015 May 9;15:476. doi: 10.1186/s12889-015-1811-5 (PMC4427931; doi:10.1186/s12889-015-1811-5)
Supplement: Additional file 2: — Zoonoses in humans and livestock in rural Cambodia – Household questionnaire. [file 12889_2015_1811_MOESM2_ESM.pdf]

Zoonoses in Humans and Livestock in Rural Cambodia
Household questionnaire

Occupation of interviewee: ___________________

Name of enumerator: _______________________________

Age: __________ **Sex:** [ ] M [ ] F

Date today: _____________________

Education: ________________________________

Person to revise this questionnaire: ___________________

1. Village/Site Name and Household Descriptors

(Village/Site Name):	(District):	(Province):
(No. of men above 15 years in the HH)	(No. of women above 15 years in the HH)	(No. of children 15 years and below in the HH)
(No. of chicken in the HH)	(No. of ducks in the HH)	(No. of other avian species in the HH, specify):
(No. of pigs in the HH)	(No. of cattle in the HH)	(No. of buffaloes in the HH)

1.1 Does the household own land for farming?

a. All farmland owned by the household ……………………………………………………………….

b. Approximate proportion of farmland owned by the household …………………………………..

1.2 Does the household have any of the following:

	Yes	No
Concrete/Brick house	☐	☐
Roof tiles	☐	☐
TV	☐	☐
Cell phone	☐	☐
Vehicle	☐	☐

Specify vehicle type: ____

1.3 What are the main crops grown by this household?

……

……

1.4 Has this household been involved in any development support (Government or NGO)?
 No
 Yes, please specify

………

2. Human Health

2.1 What main source of water do you use for drinking in this household?
 Well, untreated
 Well, treated. Specify how ....
 Nearby river or stream, untreated
 Nearby river or stream, treated. Specify how ....
 Bottled water
 Rain water, untreated
 Rain water, treated. Specify how ………………………………………………………….
 Other, specify …………………..

2.2 How many days per month do you eat the following in your household:

Pork .... Beef ....

Fish .... Poultry ....

Egg: .... Wildlife .... Other (specify): ....

2.3 Who is responsible for preparing food in this household?

………

2.4 What do you practice in this household?

Yes	No	
		Eat undercooked meat
		Feed your livestock uncooked meat waste
		Cull sick animals for consumption
		Eat animals found dead
		Wash hands with soap before and after cooking
		Wash hands with soap after handling live animals
		Keep live animals away from sleeping and food preparation areas
		Burry or burn meat waste products
		Daily collection of manure in-and outdoor
		Capture and slaughter wild animals for consumption

2.5 Do you slaughter domestic animals in this household?
 No
 Yes

2.6 If yes in Q 2.5 kindly explain how you dispose of slaughter waste products

……..

………

2.7 Has any member of the household suffered from the following signs during the last 14 days:

Yes	No	
□	□	Diarrhoea or loose stool
□	□	Bloody diarrhoea/stool
□	□	Abdominal pain
□	□	Fever
□	□	Head and/or body ache
□	□	Cough
□	□	Shortness of breath
□	□	Vomiting
□	□	Nausea

3. Livestock System

3.1 How often are there episodes of severe disease among your livestock causing high mortality?

□ At least once a year
□ Once every 2 –3 years
□ Every 5 years
□ Other, specify………………………………………………………………………………

3.2 How often are there episodes of less severe disease among your livestock i.e. respiratory diseases, gastrointestinal diseases and abortions?

□ At least once a year
□ Once every 2 –3 years
□ Every 5 years
□ Other, specify……………………………………………………………………………….

3.3 What season, if any, do outbreaks of disease in livestock usually occur?

…….

3.4 Do you use any routine animal health treatments (such as use of vaccines or anthelmintics)?

□ No
□ Yes, what & when …………………………………………………………………………………

3.5 Which member/s of the household assume the responsibility of looking after the livestock?

**<u>Pigs</u>**	**<u>Avian</u>** (chicken, ducks, geese, turkeys etc.)	**<u>Ruminants</u>** (cattle and buffaloes)
□ Employees	□ Employees	□ Employees
□ Women	□ Women	□ Women
□ Men	□ Men	□ Men
□ Children	□ Children	□ Children
□ Combination, specify	□ Combination, specify	□ Combination, specify
………………………………	………………………………	………………………………

3.6 What is the source of water used for livestock?

□ Well
□ Nearby river or stream
□ Other, specify………………………………………………………………………

3.7 How do you keep your livestock?

Pigs

 Free ranging
 Combination of free ranging and confined
 Confined with other pigs only
 Confined mixed with other species
 Combination, specify

………………………………

Avian

 Free ranging
 Combination of free ranging and confined
 Confined with the same avian species only
 Confined mixed with other species
 Combination, specify

………………………………

Ruminants

 Free ranging
 Combination of free ranging and confined
 Confined with other ruminants
 Confined mixed with other species
 Combination, specify

………………………………

3.8 If you do not keep your livestock confined kindly explain why?

………

3.9 What is livestock usually reared for?

Pigs

 Sale
 Family consumption
 To be kept for sale in an emergency
 Other, specify

………………………………

Avian

 Sale
 Family consumption
 To be kept for sale in an emergency
 Other, specify

…………………………………

Ruminants

 Sale
 Family consumption
 To be kept for sale in an emergency
 Draught power
 Other, specify

………………………………

3.10 Rank the most common trading practice when livestock are sold with 1=the most common one etc.

Pigs

Animals are taken directly to slaughter-house
Animals are taken to local market
Animals are sold to farmers in nearby villages
Animals are sold to farmers in this villages
Animals are sold to middleman
Other, specify

…………………………………………………………

Avian

Animals are taken directly to slaughter-house
Animals are taken to local market
Animals are sold to farmers in nearby villages
Animals are sold to farmers in this villages
Animals are sold to middleman
Other, specify

…………………………………………………………

Ruminants

Animals are taken directly to slaughter-house
Animals are taken to local market
Animals are sold to farmers in nearby villages
Animals are sold to farmers in this villages
Animals are sold to middleman
Other, specify

…………………………………………………………………………………………………..

3.11 Rank the most common trading practice when livestock are purchased with 1=the most common one etc.

Pigs

Purchased from local market, specify name of the market………………………………

Animals are purchased from a middleman

Animals are purchased from farmers in this village

Animals are purchased from farmers in nearby villages

Animals are purchased from government or private animal farm

Other, specify…………………………………………………………………………………

Avian

Purchased from local market, specify name of the market…………………………………

Animals are purchased from a middleman

Animals are purchased from farmers in this village

Animals are purchased from farmers in nearby villages

Animals are purchased from government or private animal farm

Other, specify…………………………………………………………………………………

Ruminants

Purchased from local market, specify name of the market…………………………………

Animals are purchased from a middleman

Animals are purchased from farmers in this village

Animals are purchased from farmers in nearby villages

Animals are purchased from government or private animal farm

Other, specify…………………………………………………………………………………

3.12 What do you usually do when you have sick livestock?

- ☐ Separate sick from healthy livestock
- ☐ Slaughter sick livestock for consumption
- ☐ Slaughter and destroy sick livestock
- ☐ Sell sick and healthy livestock
- ☐ Sell sick livestock only
- ☐ Give away sick livestock
- ☐ Vaccinate sick and healthy livestock, Specify vaccine…………………………
- ☐ Treat sick livestock, Specify treatment…………………………………………
- ☐ Do nothing
- ☐ Other, specify………………………………………………………………………..

3.13 What do you usually do if your livestock die?

- ☐ Trough away livestock found dead
- ☐ Eat livestock found dead
- ☐ Burn or bury livestock found dead
- ☐ Sell the remaining livestock
- ☐ Give away the remaining livestock
- ☐ Vaccinate the remaining stock, Specify vaccine……………………………….
- ☐ Other, specify……………………………………………………………………….

3.14 Do you notice an increase in the incidence of disease after 'new' stock is introduced into the village?

 Yes
 No

3.15 Do you see any likelihood of transmission of diseases between humans, animals and wildlife in this village?

 No
 Yes, please specify

……

3.16 What diseases with possible transmission between humans, animals and wildlife do you know of?

 None
 Some, please specify

……

……
